# Using Instructional Design Process to Improve Design and Development of Internet Interventions

**DOI:** 10.2196/jmir.1890

**Published:** 2012-06-28

**Authors:** Michelle M Hilgart, Lee M Ritterband, Frances P Thorndike, Mable B Kinzie

**Affiliations:** ^1^Behavioral Health and TechnologyDepartment of Psychiatry and Neurobehavioral SciencesUniversity of VirginiaCharlottesville, VAUnited States; ^2^Curry School of EducationDepartment of Leadership, Foundation and PolicyUniversity of VirginiaCharlottesville, VAUnited States

**Keywords:** Internet interventions, instructional design

## Abstract

Given the wide reach and extensive capabilities of the Internet, it is increasingly being used to deliver comprehensive behavioral and mental health intervention and prevention programs. Their goals are to change user behavior, reduce unwanted complications or symptoms, and improve health status and health-related quality of life. Internet interventions have been found efficacious in addressing a wide range of behavioral and mental health problems, including insomnia, nicotine dependence, obesity, diabetes, depression, and anxiety. Despite the existence of many Internet-based interventions, there is little research to inform their design and development. A model for behavior change in Internet interventions has been published to help guide future Internet intervention development and to help predict and explain behavior changes and symptom improvement outcomes through the use of Internet interventions. An argument is made for grounding the development of Internet interventions within a scientific framework. To that end, the model highlights a multitude of design-related components, areas, and elements, including user characteristics, environment, intervention content, level of intervention support, and targeted outcomes. However, more discussion is needed regarding how the design of the program should be developed to address these issues. While there is little research on the design and development of Internet interventions, there is a rich, related literature in the field of instructional design (ID) that can be used to inform Internet intervention development. ID models are prescriptive models that describe a set of activities involved in the planning, implementation, and evaluation of instructional programs. Using ID process models has been shown to increase the effectiveness of learning programs in a broad range of contexts. ID models specify a systematic method for assessing the needs of learners (intervention users) to determine the gaps between current knowledge and behaviors, and desired outcomes. Through the ID process, designers focus on the needs of learners, taking into account their prior knowledge; set measurable learning objectives or performance requirements; assess learners’ achievement of the targeted outcomes; and employ cycles of continuous formative evaluation to ensure that the intervention meets the needs of all stakeholders. The ID process offers a proven methodology for the design of instructional programs and should be considered an integral part of the creation of Internet interventions. By providing a framework for the design and development of Internet interventions and by purposefully focusing on these aspects, as well as the underlying theories supporting these practices, both the theories and the interventions themselves can continue to be refined and improved. By using the behavior change model for Internet interventions along with the best research available to guide design practice and inform development, developers of Internet interventions will increase their ability to achieve desired outcomes.

## Introduction

Emerging technologies have had far-reaching implications on global connectivity, including the availability and use of health information [1]. Given the accessibility and extensive capabilities of the Internet, it is increasingly being used to deliver comprehensive behavioral and mental health interventions. Their goals are to change user behavior, reduce unwanted complications or symptoms [2], and improve health status and health-related quality of life [3]. The Internet (and other technologies) offers the potential to provide efficient, interactive, tailored, and readily accessible health interventions [2].

### Internet Interventions

Results of meta-analyses of Internet-delivered interventions show evidence of their efficacy for addressing a broad range of behavioral and mental health concerns [4-6]. Internet interventions have been found efficacious in addressing a variety of behavioral and mental health problems, such as insomnia, nicotine dependence, obesity, diabetes, depression, and anxiety [7-14]. Yet, despite the existence of many Internet-based interventions, the design of these interventions is widely variable, and there is little research to inform their design and development.

A model for behavior change in Internet interventions has been published [2] to help guide the development of Internet interventions and to help predict and explain behavior changes and symptom improvement through the use of these programs [2]. With this model, Ritterband and colleagues argue for grounding the development of Internet interventions within a scientific framework—a framework that explicitly identifies the importance of user characteristics, environment, intervention content, level of intervention support, and targeted outcomes. More discussion, however, is needed regarding *how *the design of the program should be used to address these very issues.

### Instructional Design Defined

While there is little research on the design and development of Internet interventions, there is a rich, related literature in the field of instructional design (ID). The term instructional design can be considered in three specific contexts. First, as a *science*, ID is concerned with how to help people learn more effectively. It includes research and theory about instructional, motivational, and behavioral learning strategies and the process models for designing and implementing instructional programs [15-18]. Second, as a *field of practice*, ID includes professional instructional designers working with teams of individuals to create detailed specifications for the development, design, implementation, evaluation, and maintenance of learning products [19]. These individuals make up part of the primary stakeholder groups for an intervention, together with other individuals who have an investment, or stake, in the success of the target population of learners (program users). Stakeholders include program developers, content experts, learners from the target population, and those affected by the program outcome. Finally, ID, as a *process, *(see Table 1) employs process models to guide the systematic development of instructional specifications drawing on learning, instructional, motivational, and behavioral theory to ensure the quality of instructional strategies.

Additionally, the ID process allows the translation of these theories into design principles that guide the development of the instructional product. This paper is focused on using ID as a systematic, reflective, and iterative process in the development of Internet interventions.

**Table 1:** The instructional design (ID) process: terms and definitions.

Instructional design	The systematic development of learning programs using theory to ensure the quality of instruction. It is the entire process of analysis of learning needs and goals, and the development of a delivery system to meet those needs.
ID process model	Prescriptive models that describe a systematic set of activities and steps involved in the planning, implementation, and evaluation of instructional programs.
Developmental research	The systematic study of designing, developing, and evaluating instructional programs, processes, and products that must meet the criteria of internal consistency and effectiveness.
Learners	The target population of an intervention: the individuals for whom the program or intervention is created.
Internet interventions	Internet-delivered, interactive, multimedia behavioral treatments often based on effective face-to-face interventions. Typically self-guided, highly structured, personalized, and tailored to the user to provide follow-up and feedback.
Stakeholders	Individuals who have a stake in the success of the target population of learners (program users). Stakeholders include program developers, content experts, learners from the target populations, and those affected by the program outcome.
Formative evaluation	The iterative process of tryout and revision of instruction and activities during development of the intervention before the actual implementation.
Needs assessment and analysis	Considers gaps between “what is” and “what should be” or “actual behaviors” versus “optimal behaviors.” Each gap is considered a need. A needs assessment or analysis is a process for determining how to close gaps. It involves identifying the required attitudes, behaviors, skills, and knowledge to meet needs.
Instructional goal	Formulated from the identified needs in the needs analysis. Instructional goals relate logically and persuasively to the documented performance gaps identified in the needs analysis.
Task analysis	Performed to identify the tasks required to reach the goals. This is an analysis of the content required for the desired instructional outcomes.
Learning objective	Written to specify exactly what the learner must do, know think, or feel as a result of completing the instruction. Objectives provide a framework for assessing and evaluating the extent to which learning is taking place.
Affective objective	Objectives that involve attitudes, emotions, and values.
Cognitive objective	Objectives and tasks related to information, knowledge, problem solving, and other intellectual aspects of learning.
Psychomotor objectives	Objectives that require the use of physical capabilities and activities, such as performing, manipulating, and constructing tasks.

### Internet-Delivered Interventions and Instructional Design

During the past decade, educational researchers have focused on conducting design and development research to advance the practice of instructional development [15,20,21]. Studying the design and development process of an innovative instructional product can help developers better understand how to apply theoretical frameworks to the development process [22]. The same case can be made for studying the design and development of Internet-delivered interventions.

Developmental research in education seeks to create knowledge grounded in data systematically derived from practice [20]. By focusing specifically on the design and development process of creating Internet interventions, we can gain knowledge of the best practices and methods, and can develop more efficient models and frameworks for creating Internet interventions. Incorporating the ID process into the design and development of Internet interventions allows for continued testing and refinement of the theories that guide development and improvement in the resulting interventions. This is the essence of design-based research; theory dictates the design principles. Employing ID process during the design of Internet-delivered interventions allows the programs to be evaluated for efficacy while also testing and refining the design principles that initially informed development of the intervention [23].

At their core, Internet interventions are innovative programs designed to teach skills, increase knowledge, and change behaviors, symptoms, or other targeted attributes. A majority of Internet interventions target specific behavioral, psychological, motivational, or health education outcomes, or a combination of these. Each intervention is implemented within its own context in which individuals from the target population (called learners or users) will use and (ideally) benefit from the program. It is important to clarify at the outset that the term learner or user is used to identify Internet intervention participants given their enrollment in a program designed to bring about change. These learners, however, are considered in different ways within the design process and the intervention itself (eg, patient or caregiver; child or parent; or children, teens, adults, or seniors). Although Internet-delivered intervention participants may not think of themselves as learners, they share the fundamental characteristic with learners of any intervention or involvement in a program with the aim of change, improvement, or advancement. This, together with the similarity of learning purposes for Internet-delivered health interventions and other types of instructional interventions, makes the ID process an excellent fit for the design and development of Internet-delivered interventions.

### Models of Instructional Design

ID models can be used prescriptively to describe a systematic set of activities and steps involved in the planning, implementation, and evaluation of instructional programs. Using ID process models has been shown to increase the effectiveness of learning programs in a broad range of contexts, including both online and face-to-face formats [19,24-27]. ID models prescribe a systematic method for assessing the needs of learners to determine the gaps between current attitudes, behaviors, and knowledge and the desired outcomes [28]. The ID process guides designers to focus on the needs of learners, taking into account their prior knowledge [29]; set measurable learning objectives or performance requirements; design instructional strategies based on appropriate theory; assess users in a way that results in meaningful outcomes [30,31]; and use cycles of formative evaluation to ensure that the intervention meets the needs of all stakeholders.

**Figure 1:**
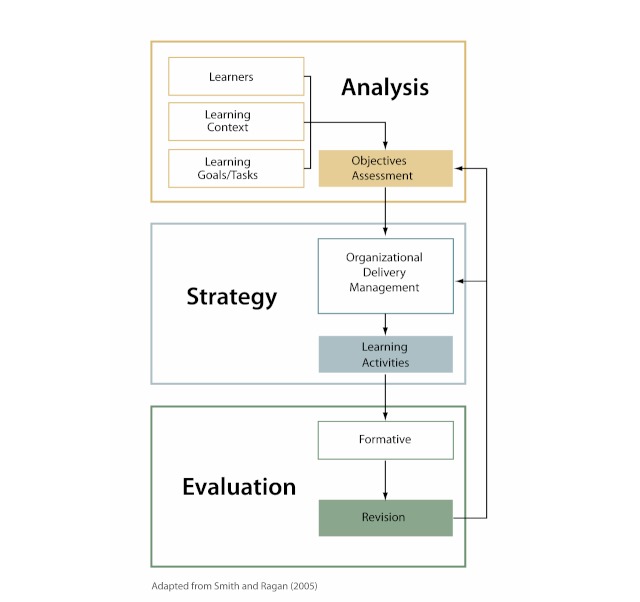
Smith and Ragan’s instructional design process model.

We describe two ID models to convey the breadth in models from simple to elaborate, showing how ID models ultimately focus on similar activities. On one end of the complexity spectrum, Smith and Ragan [27] present what they call “a simple or common instructional design model” (see Figure 1) that focuses on three key activities or phases of the ID process: *analysis*, *strategy*, and *evaluation*. Analysis activities consist of assessing learners and learning contexts and developing learning goals. Strategy activities focus on design, organization, and delivery of instructional components. Evaluation activities focus on formative tryout of instruction to allow for revision before implementation.

Although the model is presented as linear, its authors point out that phases often happen concurrently, and considerations in one phase may (and often do) overlap with those in another phase. An important aspect of the ID process is that it is iterative. Formative evaluation begins during the first cycle, with “member checking” of the needs assessment, and continues throughout development. The results of the formative evaluation are used to make revisions to the intervention. The cycles repeat, with further evaluations guiding further revision.

**Figure 2:**
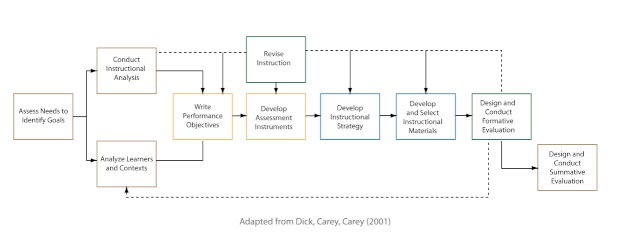
The Dick and Carey Systems Approach Model for Designing Instruction.

In contrast to the simplicity of Smith and Ragan’s ID model [27], Dick and Carey’s Systems Approach Model for Designing Instruction (see Figure 2) presents a more complex ID model. This model displays more of the specific ID activities that take place [19], yet each of these activities can be mapped to the analysis, strategy, and evaluation components outlined in the Smith and Ragan model [27]. The key ID activities of analysis, strategy, and evaluation are described in detail below.

### Analysis

In the analysis phase, the focus is on the targeted learner population (intervention users); the context and environment in which the learning (intervention) occur; identification of learning (intervention) goals and objectives; and the learning (intervention) tasks themselves. A needs assessment is performed to collect the information used in the analysis phase. Needs assessment and analysis considers the gaps between “what is” and “what should be.” A needs assessment is a process for determining how to close a learning or performance gap [32] and involves identifying the important needs and how best to meet them.

A particularly useful and relevant needs assessment approach, the discrepancy model of needs assessment, examines the gaps, or differences, between individuals who perform desired behaviors and those who do not [27]. For example, when a successful intervention requires learners to perform a behavior (take a medicine), use knowledge (recognize a skin problem), or show an attitude or belief (perceive one’s own risk), the differences between what ideal performers do, think, and feel are compared with what learners in the target population are actually doing, thinking, and feeling. Once gaps have been identified, the causes of the gaps can be studied and quantified. This information then shapes development of the learning or intervention tasks designed to achieve the intervention goals.

Internet intervention development is often based on effective face-to-face treatments where specific desired learning and performance outcomes have already been clearly identified and tested [33]. That is, Internet intervention developers frequently borrow from the goals identified in face-to-face treatments. Using the discrepancy approach to needs assessment, Internet intervention developers can focus on the causes of the gaps between what their target population is currently doing (knowing, thinking, or feeling) and what they should be doing to achieve the targeted outcome. Each gap discovered in the analysis is considered a need.

The discrepancy approach can also be used in instances where optimal behaviors have not been identified or are not known. In this case the optimal, or ideal, behaviors must be identified as part of the needs assessment process by collecting information from the target learners. This includes collecting information from those who have successfully achieved the desired outcome, as well as those who have not. Data are collected to inform developers about the level of awareness of the problem or condition of interest, the common symptoms, how risk is typically managed, and the level of adherence to preventive behaviors. As the data are analyzed, the attitudes, behaviors, skills, and knowledge of the successful individuals are identified and quantified as the optimal set of behaviors, skills, and knowledge. The differences between this ideal set and the unsuccessful set can then be identified as gaps or needs.

In the ID process, identified needs are then formulated into goals. It is critical that goals relate logically and persuasively to the documented performance gaps identified in the needs analysis. The importance of considering learner-based needs cannot be overstated. Programs must target the needs of the identified population in order to be successful [19,28]. Instructional and goal analysis (systematic methods for analyzing goals to identify the required knowledge, skills, behaviors, and attitudes to meet them) is used to categorize and prioritize goals, based on the kind of learning that will occur, into a series of specific measurable and observable objectives.

Objectives perform several critical functions in the design of instruction, including guiding designers toward the appropriate focus for instruction, and selecting activities and resources that facilitate effective learning [29]. Objectives also provide a framework for assessing and evaluating the extent to which learning is taking place and play an important role in guiding the learner by identifying the skills and knowledge to master [27,29].

During the analysis phase, the designer also considers the environment and context in which learning will take place. The environmental approach to analysis is based on three environmental domains: physical, social, and institutional [34]. Physical concerns are those related to the physical environment in which the intervention will be used. Social concerns refer to the learners and their social connections and networks, including those that will influence the learning experience. Institutional considerations affect any institutional goals held by the sponsoring organization and help define the dissemination and use of the program. Each of these domains informs decisions that affect a learner’s ability and willingness to access and use the program. By working closely with members of the targeted population at the early stages of design and development, designers can fully consider the domain factors most relevant for creation of programs that reflect and address the needs of learners, as well as aligning with the domains in which the programs will be used.

Task analysis, the next step in the ID process, is conducted to identify the actual tasks required to reach the goals identified in the needs analysis. This second analysis considers the content required for the desired instructional outcome [29]. Although there are many ways to analyze tasks, most ID models provide a scheme for classifying information into discrete categories [15]. The objectives and tasks typically fall into one of three domains, or categories: cognitive, psychomotor, and affective [29]. The cognitive domain includes objectives and tasks related to recall of information, development of conceptual knowledge, application of knowledge to problem solving, and other intellectual aspects of learning. The psychomotor domain includes skills that require the use of physical capabilities and activities, such as performing, manipulating, and constructing tasks. The affective domain includes objectives targeting attitudes, emotions, and values. Thoughtful consideration of the learning experience, including the cognitive, psychomotor, and affective factors involved in that experience, will increase the likelihood that learners can successfully develop the desired knowledge, skills, behaviors, or attitudes.

In summary, the analysis phase of the ID process involves two sets of analyses. The first analysis identifies the learners (intervention users), the learning (intervention) objectives, and the environment in which the learning (intervention) occurs. The second analysis considers the content, type of tasks, and learning experiences required to meet learning objectives.

### Strategy

To guide creation of the instructional activities, the strategy phase of the ID process is informed by and draws upon tested theories. Theories are drawn from a range of fields including education and learning psychology, behavior change, and motivation [16-18,35-38]. Theories that have been widely applied include cognitive learning [18,35,39,40], information processing [41], and multimedia learning [42]. The structure and type of learning required by each objective influences which learning theories are most applicable. The selection of instructional strategies is also clearly influenced by the analysis of the content; that is, the determination of domain (ie, whether the target is a behavior, skill, knowledge, or attitude), as well as the analysis of the tasks that make up the desired performance. For example, if the intervention aim is to teach the users a rule (eg, to get out of bed if they have not fallen asleep in 20 minutes as part of an intervention for adults with insomnia), the developer first recognizes that this knowledge acquisition falls in the cognitive domain, and then considers the cognitive skills that lead up to and support the rule application (eg, conceptual understandings, which are in turn supported by information recall). Developers would then turn to cognitive theories to help inform their development of the learning activities designed to facilitate learning to apply this rule. Different types of learning tasks require substantially different levels of cognitive effort and different kinds of learning conditions [24,27,29].

In this strategy phase of the ID process, the focus is on designing the learning activities that will best serve the specific set of learners for whom the program is being developed. Learning activities refer to learning experiences that involve informational content and designed experiences in which learners act on content in specific ways. Consideration is given to each specific objective and how best to actively engage learners with the learning experiences in order to obtain the desired result. The crafting of instructional strategies is considered the most crucial step in the ID process; it is the step that can contribute the most to making instruction successful [29].

When selecting instructional strategies, designers also need to consider and select the media and methods best suited to deliver the desired experience. Smith and Ragan’s model [27] (see Figure 1) highlights three key categories in the strategy phase: organization, delivery, and management. Organizational strategies focus on how instruction will be sequenced and presented. Delivery strategies are concerned with the instructional media that will be used and how learners are grouped. For example, learners may be grouped by level of prior knowledge, attitudes on the topic, skill abilities, motivation level, or presence of specific symptoms. In selecting the appropriate media elements used for the learning activities (eg, text, audio, graphics, and animation), developers should evaluate the motivational appeal and ability of each element to support learners in recalling prior knowledge, providing new learning stimuli, activating responses, providing feedback, and encouraging practice and transfer [16,19,43]. Finally, management strategies focus on the scheduling and implementing of instruction.

In sum, strategy activities are critical to the ID process. They include the design and development of the actual learning and behavior change activities that will be used to help achieve the objectives and offer designers the opportunity to draw on strategies that have previously been shown to be effective within other specific contexts. The strategy phase also focuses on the organization or sequencing of learning activities, and the media and delivery methods used to engage learners with the materials.

### Evaluation

Formative evaluation refers to the iterative process of tryout and revision of activities or content during development before the actual implementation [44]. The evaluation phase tests the assumptions made in the analysis and strategy phases. The purpose of formative evaluation is to ensure that the goals of the instruction are being achieved and to revise the program as needed before implementation. Formative evaluation requires a plan for determining the extent to which learning is taking place. It involves trying out learning activities with members of the learning group. Evaluation instruments are used to assess the learner’s mastery of the objectives.

Formative evaluation ideally takes place at all stages of the ID process. In fact, Dick et al incorporate a formative evaluation element into each stage of their ID process model [19] where draft versions of the instruction are examined and then revised as needed. Formative evaluation during the earliest stages of the ID process (even needs assessments can be member checked and refined for fit by stakeholders) can help determine whether the learning goals and objectives have been correctly identified, and whether assumptions made about learners and learner characteristics hold true. This helps prevent valuable time and resources from being wasted on components that are not effective. Using an ongoing formative evaluation approach, and revising the program based on findings, allows developers to identify weaknesses that can be corrected before full-scale implementation [19,24,29,44]. It also increases the probability that the program, when implemented, will produce the desired learning and performance outcomes. Failure to conduct formative evaluation throughout all stages of the ID process misses opportunities for identification of problem areas and increases the possibility that learners will not achieve the intended goals despite considerable investment of resources.

## Integrating ID Process Into the Design and Development of Internet Interventions

**Figure 3:**
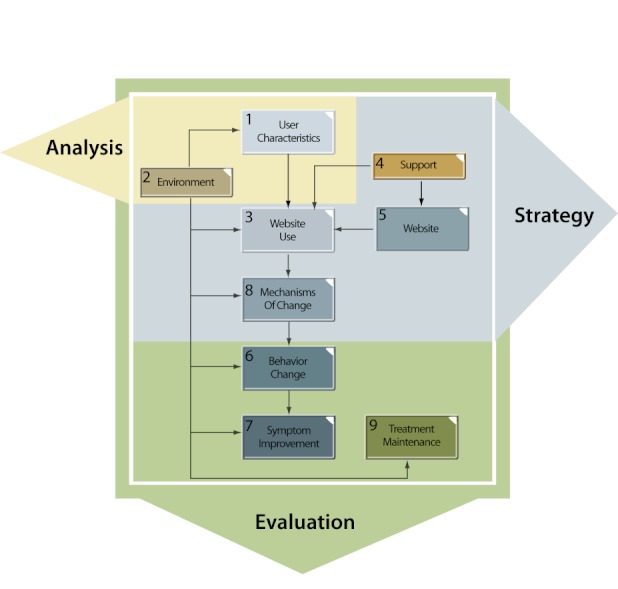
Instructional design process model for behavior change in Internet interventions.

Ritterband and colleagues [2] described a behavior change model for Internet interventions (see Figure 3 for a high-level representation of the Internet intervention model juxtaposed with the ID model) that consists of nine nonlinear steps: (1) the *user*, influenced by (2) *environmental factors*, affects (3) *website use *and adherence, which is influenced by (4) *support *and (5) *website *characteristics. *Website use *leads to (6) *behavior change *and (7) *symptom improvement *through various (8) *mechanisms of change*. The improvements are sustained via (9) *treatment maintenance *or relapse prevention. Each section of this behavior change model can be clearly connected to the ID process.

### User Characteristics and Environmental Factors

Ritterband and colleagues [2] identify seven areas of user characteristics that are congruent to the analysis phase of the ID model, including disease, demographics, traits, cognitive factors, beliefs and attitudes, physiological factors, and skills. Using an ID approach focuses the needs assessment on discrepancies between the user’s *desired *behaviors, skills, knowledge, or attitudes and their *current *behaviors, skills, knowledge, or attitudes. Identifying these discrepancies accomplishes two important functions: (1) it attempts to quantify the current state of affairs with regard to the target population so that progress toward meeting goals can be accurately measured [24], and (2) it allows very specific learning and performance objectives to be crafted for the intervention that are based on the desired outcomes.

Environmental factors are also considered in the analysis phase of the ID model. As in Ritterband and colleague’s model [2], environment from the ID perspective is composed of multiple influences, and the focus on environment is holistic. Assessment methods are used to construct an environmental snapshot of how the program will be used by the learner from the physical, social, and institutional contexts of the learning environment. When using the ID model, designers can identify potential program supports and barriers and design the program accordingly. For example, through a needs assessment, designers of an Internet intervention targeting users with negative attitudes toward school are alerted that labeling recommended intervention activities as homework or assignments may present barriers for these users. The ID model also allows designers to leverage environmental factors that influence user characteristics. By systematically identifying the environmental factors, along with the characteristics of the user population, program designers can choose the most relevant motivation, learning, and behavior change theories for their set of learners and learning objectives based on the research literature. This, in turn, will affect the type of instructional strategies that are planned in the strategy phase.

### Website Use, Website Characteristics, Support, and Mechanisms of Change

The website use, website characteristics, support, and mechanism of change components of the Internet intervention model all map to the strategy phase of the ID process model. All phases of the ID process are interrelated, so the findings and assumptions made in the analysis phase greatly affect the decisions made in the strategy phase. The emphasis in the strategy phase is on crafting the instructional strategies used to reach the desired goals. This focus underscores the importance of design-based research. Using a design–study approach, developers of Internet interventions tap theory for instructional strategy design guidelines that are then tested along with the instructional components. Thus, knowledge is created in the refinement of theories of both design or development and instruction in the context of Internet-delivered interventions.

There are several specific challenges in considering instructional strategies in the context of Internet interventions. Opportunities to leverage technologies to build activities that support and promote learning are often not fully considered in favor of less expensive and more quickly produced solutions. For example, text-based delivery of learning is recognized as an important method, yet, before a text-focused solution is selected as most appropriate, it must be considered in relation to the target population of learners, their prior experiences and attitudes, and their reading levels. When text is used, learners can be grouped based on reading level or supported by providing narration so those with lower-level reading skill have the choice of listening instead of reading. Video clips, animation, audio segments, and images can all be integrated with text to support meeting learning objectives.

Multimedia learning and using specific interactions to affect motivation, skill building, and behavioral change demand instructional strategies that move beyond text on a screen. These methods can be particularly appropriate when an intervention includes psychomotor skill building. Modeling, through the use of videos or animation of specific skills, using a variety of models and conditions can be more effective than reading about the activity [18,42]. Additionally, modeling typical beginner mistakes, while providing specific feedback on why problems occur and ways to correct or prevent them, can help learners gain mastery [30].

Motivation, or the action of an individual to select and sustain a behavior, is another theory area from which designers of instructional strategies can find significant guidance. Motivation theories help focus attention and support affirmative answers to questions such as “Can I do this task?,” “Do I want to do this task?,” and “Will I continue to do this task?” Self-efficacy [45], expectancy-value [17], self-determination [46,47], and self-regulated learning theories [47] can all provide designers with practical and applicable programmatic supports that can help learners meet learning objectives and help sustain adherence to intervention use.

The strategy phase also focuses on organizing and sequencing learning activities in ways that will best help users meet identified program goals. Strategy activities include selecting media and methods for delivery of the instruction, and managing and supporting the implementation of the instructional strategies. In each of these areas, the primary concern is how best to engage the learner to reach agreed-on goals.

### Behavior Change, Symptom Improvement, and Treatment Maintenance

The formative evaluation component of the ID model is the evaluation of program components completed while the intervention is being formed. This type of evaluation provides evidence for how well designers have reached their goals and allows modification of the program before it is fully implemented. Behavior change, symptom improvement, and treatment maintenance or relapse prevention components of the Internet intervention model align with the evaluation phase of the ID process. During formative evaluation activities, results of the needs assessment, instructional goals, objectives, and strategies are evaluated with content experts and members of the target population to explore whether the assumptions made, the strategies employed, and the learning activities developed actually result in the desired outcomes.

To determine the extent to which goals are being met, designers develop and implement a plan for assessing outcomes from their intervention. Evaluation of instruction typically considers outcomes at four levels: (1) learner reactions, (2) learning achievement, (3) transfer of learning, and (4) organizational results [26]. Formative evaluation focuses on the first two levels [24], while summative evaluation (program evaluation that occurs after implementation) may focus on all four levels. The first level, learner (user) reactions, refers to the attitudes and preferences of the learners toward the learning intervention. This is the extent to which learners like or dislike the learning activities, or find the activities to be satisfactory, effective, and useful. The second level, learning (intervention) achievement, refers to how well learners perform on objective measures of learning. This is often tested with pre and post tests to determine the extent to which learners have mastered goals via change in attitudes, knowledge, skills, or behavioral intentions.

During the revision stage of formative evaluation, the data that have been collected are analyzed and used to operationalize a set of revisions to the intervention. The ID process includes setting standards and criteria to guide revisions. This includes criteria for examining the data that have been collected; criteria for organizing and summarizing the data; and criteria for prioritizing which sources of data are most relevant to the revision efforts [44]. Prioritizing and deciding how to implement revisions is typically the most challenging for designers. It can be relatively straightforward to identify a problem area but less clear what should be the appropriate revision or refinement of the instruction. For example, an evaluation of learners on how well they like an intervention and find it satisfactory and useful (level 1) returns negative results for one set of learners but positive results for other sets. Designers need to think about how the learners are being grouped and whether the set of learners with the unsatisfactory experience share characteristics that can help inform program revisions (eg, gender differences, differences in prior knowledge, differences in symptoms, or age-related differences). Designers also need to consider the selected theoretical underpinning and whether it is appropriate for the subset of learners with negative outcomes. Designers should investigate whether additional needs within a subset of the user population can be identified and supported by adding appropriate objectives, content, and instructional strategies, thus making the intervention satisfactory, useful, and relevant to all user groups. It is often necessary to reconsider the previous stages of analysis and strategy to determine how best to revise the instruction [19].

By conducting preliminary testing of the Internet intervention with users from the target population, designers will be able to measure the extent to which they have reached their objectives and to further refine goals or strategies to best reach the desired outcomes. All elements of the Internet intervention model are incorporated because formative evaluation is conducted at each phase of the ID process to confirm the assumptions made in that phase. Another important aspect to note is that, because the ID process is highly interconnected, all elements of the model overlap considerably. For example, while this mapping shows behavior change, symptom improvement, and treatment maintenance as mapping only to the evaluation phase of activities of the ID process, there is also a clear connection to both analysis and strategy activities.

## Application

Here are several examples to help convey the process of incorporating ID into the development of Internet interventions. These examples are broken down into the analysis, strategy and evaluation phases.

### Analysis Phase

**Figure 4:**
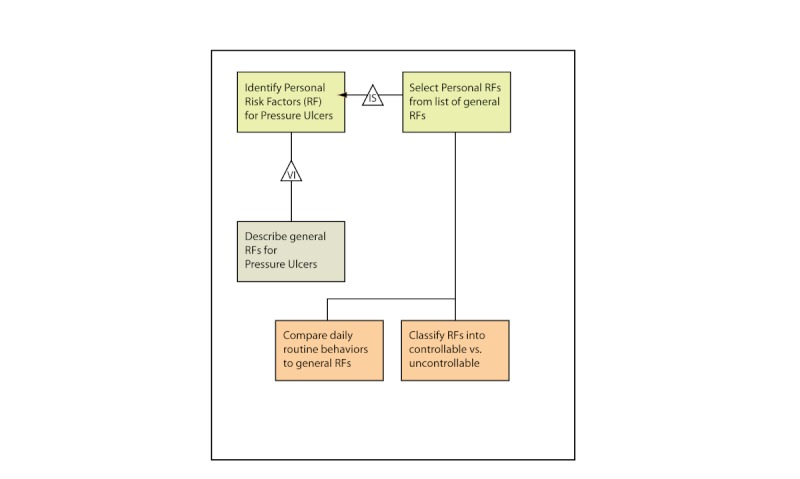
Example of instructional curriculum mapping. Program objective: core level. IS = intellectual skill; VI = verbal information.

Instructional curriculum mapping is an ID method that uses flowcharting to illustrate instructional relationships within a program [48]. An example of the use of instructional curriculum mapping flowcharts is seen in the development of iSHIFTup (Internet Skin Health Intervention For Targeted Ulcer Prevention), an Internet-delivered intervention designed for adults with spinal cord injury to prevent serious pressure ulcers (funded by the Commonwealth Neurotrauma Initiative, Virginia, USA). Within iSHIFTup, instructional curriculum mapping flowcharts were used to show how objectives map to instructional sequences. Figure 4 shows a visual representation of the relationship between an objective for learners in iSHIFTup “to identify personal risk factors for pressure ulcers” and the skills and attitudes required to meet the objective. In this example, the objective skill is within the cognitive domain. The desired outcome is that learners be able to identify their own risk factors. To do this, learners first acquire the skills to describe general risk factors, then compare their own behaviors with these factors and classify their risk factors. Once learners have mastered these sequences, they are able to identify personal risk factors for pressure ulcers.

**Figure 5:**
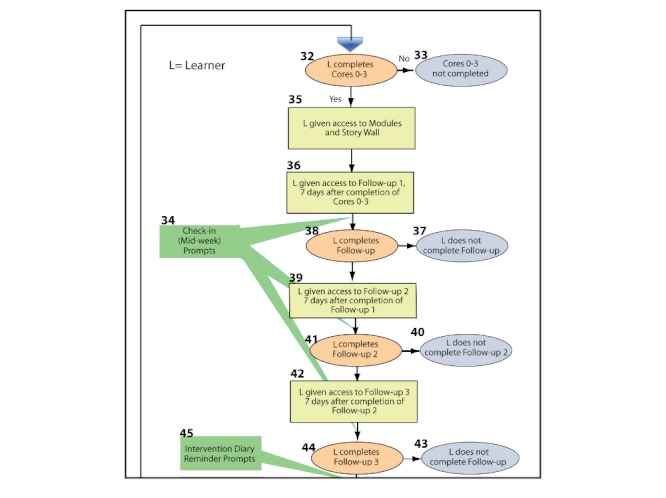
Example of instructional curriculum mapping. Program overview: sequence.

Figure 5 (a small section of a larger, fully developed iSHIFTup flowchart) graphically shows learners’ movement through the intervention from their first contact with the program to completion of all required intervention components. Flowcharting allows communication of the complex timing of events used in Internet interventions. Using these systematic methods allows visualization of the entire intervention and facilitates discussion with all stakeholders (including members of the target population of learners) to help reach consensus on the process. Using flowcharts to envision the whole system as it is being designed supports coordination between designers, developers, and those who will implement the program [19,27]. It also allows for a common language and general procedure among the stakeholders. These flowcharts, and the written plans that accompany them, are results of the ID method. They aid the development team in the process of iterative review and revision of work in a coordinated and systematic manner.

Another example of ID process in the analysis phase is the inclusion of learners from the target population in the design of the intervention. Including members of the group who will use the program in its design is critical to the success of the intervention. For example, in the design of iSHIFTup, 10 individuals (8 with spinal cord injury and 2 caregivers of patients with spinal cord injury) partnered with the development team. In addition to the 10 target user members, the development team consisted of content experts in the area of Internet-delivered interventions, pressure ulcers, and spinal cord injury medicine (clinical psychologist, rehabilitation physician, wound specialist, physical therapist, and occupational therapist), an instructional designer, a graphic artist, and a Web programmer. Collaboratively, the team identified program goals, content, and functional requirements for the intervention. Individuals with spinal cord injury shared their real-life stories about the challenges of keeping their skin healthy and maintaining preventive behaviors. They shared critical information about living with pressure ulcers and identified which coping strategies had (and had not) worked for them. Members of the user population also reviewed intervention content and instructional activities throughout the design and development of the program. They gave feedback that was incorporated into program revisions, and later reviewed the revised the content. This collaboration serves to ensure relevance and acceptability to the targeted learners (users) of the intervention.

### Strategy Phase

A comprehensive set of theory-driven instructional strategies has been recommended for health education [49]. A condensed version of Gagne’s events of instruction [43] is used as a starting point, or framework, on which the health belief model [50], social cognitive theory [18], and diffusion theory [51] are drawn as key health behavior change theories to inform instructional strategy development. Specific instructional practices based on these theories are advanced for each of Gagne’s instructional events. For example, in the recommended strategy for Gagne’s event *provide learner guidance*, social cognitive theory suggests using trustworthy, knowledgeable modeling to demonstrate desired behaviors and social modeling to develop self-protective skills. For example, a series of photographs or illustrations of a trusted, competent person performing preventive or protective behaviors could be used. Diffusion theory suggests using trustworthy and knowledgeable opinion leaders from the target population to speed the diffusion process. An example of an instructional strategy informed by both of these theories would be a video of a recognized opinion leader (eg, well-known athlete, musician, or actor), who is identified with the target population, performing desired behaviors, such as checking blood glucose levels before driving.

**Figure 6:**
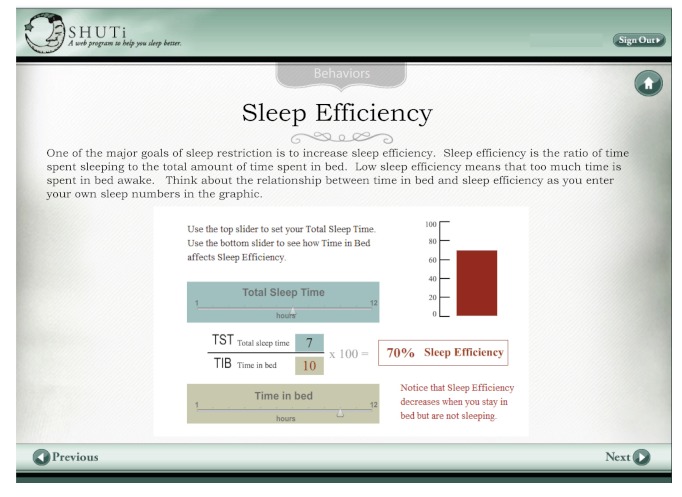
SHUTi (Sleep Healthy Using The Internet) sleep efficiency interaction screen.

Another example of using ID process during the strategy phase can be seen in SHUTi (Sleep Healthy Using The Internet), an Internet intervention for adults with insomnia [7]. One of the learning goals in this intervention is that users “recognize the relationship between *Time in Bed *and *Sleep Efficiency*.” This relationship has been identified as a conceptual understanding required to apply the behavioral rules of sleep restriction. Sleep efficiency is defined as the mathematical calculation of *total sleep time *divided by *time in bed*, multiplied by 100. In SHUTi, learners are cued (using highlighting and color) to move sliders to set and subsequently increase and decrease their total sleep time and time in bed to graphically see the relationship between the two. For example, as time in bed (while not asleep) increases, sleep efficiency decreases; and as time in bed approaches total sleep time, sleep efficiency increases (Figure 6).

This interactive, user-controlled activity was selected as an ideal way to engage learners, based on their characteristics (above-average education, high motivation, and comfort with technology), the content being introduced (cognitive domain, making connections, and intellectual skill), and type of learning goal (to recognize the relationship between time in bed and sleep efficiency and apply it to their own situation). Several learning theories were applied in crafting this solution. Guided discovery e-learning architecture [52] was selected in which knowledge construction is the learning goal and high interactivity is used to guide learners to specific goals such as making connections and identifying relationships. Multimedia learning theory suggests that people learn better when graphics are used to show relationships [53]. Here, the quantitative relationship between time in bed, total sleep time, and sleep efficiency is shown graphically. By adding interactivity in the form of slider bars, it becomes a transformational graphic that depicts changes over time [42]. The theory of planned behavior [54] is used to encourage learners to consider their own behaviors (time in bed) and intentions.

These examples demonstrate the ID process of drawing on learning theory and applying it to the specific learning context in which the intervention is being used. Design-based research, which blends empirical instructional research with the theory-driven design of learning environments [55], is an important methodology for discovering which learning and behavior theories work in Internet interventions and under what conditions. This can lead to sharable theories that help communicate relevant implications to developers of Internet interventions [23,55]. Using this type of design-based research approach focuses on methods that document processes of enactment to outcomes of interest.

### Evaluation

The final example focuses on formative evaluation and revision of instruction as part of the evaluation phase of ID. This example is an Internet-delivered intervention designed for pediatric encopresis (UCanPoopToo) [8,33]. In this program, learners (parent and child dyads) using the intervention took part in formative evaluation activities. Analysis of the findings according to agreed-on criteria revealed that parents using the program could be further supported by including a self-assessment to determine whether their children had mastered each unit of instruction. Once this gap had been recognized, additional learning goals were identified to support parents in assessing their children’s mastery. Self-regulated learning theory [47] was used to inform the design of self-assessments of the child’s content mastery and to allow parents to reflect on and adapt their children’s learning processes toward the learning goals.

**Figure 7:**
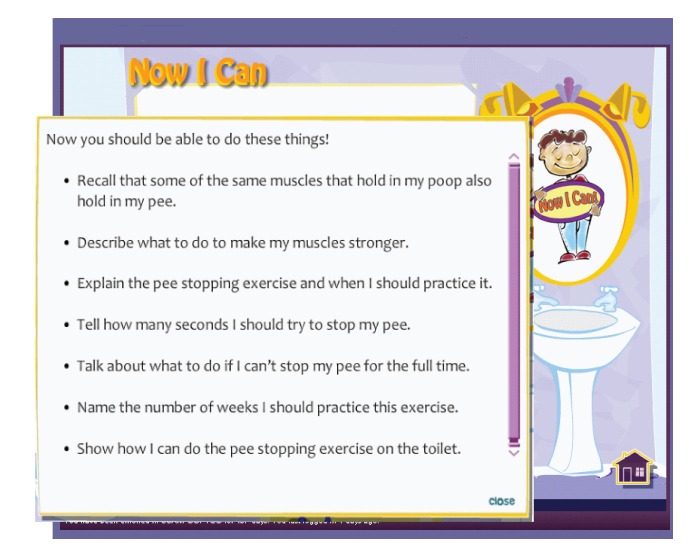
Now I Can screen from UCanPoopToo.

In the revision phase of the formative evaluation, the design team used core objectives as a starting point for developing the self-assessments. The result (see Figure 7) is a self-assessment at the end of each unit of instruction called *Now I Can*. Each Now I Can screen describes specifically what the child should be able to do at the end of the instruction. In current versions of the program, parents complete Now I Cans with their child to assess whether their child has mastered the skills in that core of instruction.

**Figure 8:**
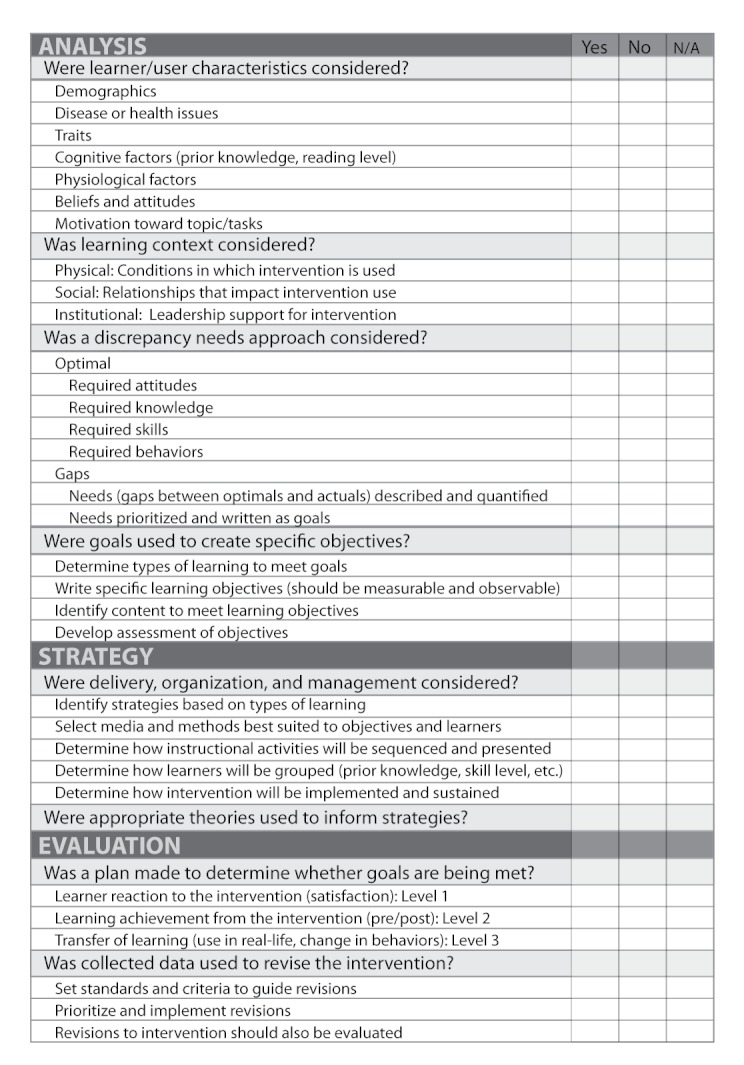
Internet intervention instructional design check list.

### Conclusion

Developers of Internet interventions often struggle with the question of whether adding a particular feature to an intervention, such as a game, a simulation, or animation, is a good or bad idea. This, however, is not the salient question to consider. Instead, given the concepts of the ID process set forth here, designers of Internet-delivered interventions are encouraged to take a learner-centered, needs-based approach and to consider how *all *technology features (eg, text, graphics, interactivity, video, and games) can be used in ways to best meet the needs of learners. It is not a simple question of whether to include a feature, but is instead a broader, more complex question of what theory-based learning activities best support a specific set of learners given their own characteristics and learning environment to meet an identified set of measurable objectives.

The ID process offers a proven methodology for the design of instructional programs and should be considered an integral part of the creation of Internet interventions. To support researchers, an Internet intervention ID check list has been created and included to use in developing new Internet-delivered interventions (see Figure 8). By providing a framework for the design and development of Internet-delivered interventions and by purposefully focusing on the design, development, and the underlying theories supporting these practices, both the theories and the interventions themselves will continue to be refined and improved. By using the behavior change model for Internet interventions along with the best research available to guide design practice and inform development, developers of Internet-delivered interventions will increase their ability to help users achieve the desired outcomes.

## Abbreviations

ID: instructional design
iSHIFTup: Internet Skin Health Intervention For Targeted Ulcer Prevention
SHUTi: Sleep Healthy Using The Internet
